# A qualitative evaluation of general practitioners’ views on protocol-driven eReferral in Scotland

**DOI:** 10.1186/1472-6947-14-30

**Published:** 2014-04-09

**Authors:** Matt-Mouley Bouamrane, Frances S Mair

**Affiliations:** 1 University of Aberdeen, Institute of Applied Health Sciences, Aberdeen, Scotland, UK; 2 College of Medical, Veterinary and Life Sciences, Institute of Health & Well-Being, Glasgow, Scotland, UK

**Keywords:** *(Mesh)*, Perioperative nursing, Medical informatics applications, Information systems

## Abstract

**Background:**

The ever increasing volume of referrals from primary care to specialist services is putting considerable pressure on resource-constrained health services while effective communication across fragmented services remains a substantial challenge. Previous studies have suggested that electronic referrals (eReferral) can bear important benefits for cross-organisational processes and patient care management.

**Methods:**

We conducted 25 semi-structured interviews and 1 focus group with primary care providers to elucidate General Practitioners’ (GPs) perspectives on information management processes in the patient pathway in NHSScotland, 1 focus group with members of the Scottish Electronic Patient Record programme and one interview with a senior architect of the Scottish Care Information national eReferral System (SCI Gateway). Using Normalisation Process Theory, we performed a qualitative analysis to elucidate GPs’ perspectives on eReferral to identify the factors which they felt either facilitated or hindered referral processes.

**Results:**

The majority of GPs interviewed felt that eReferral substantially streamlined communication processes, with the immediate transfer of referral documents and the availability of an electronic audit trail perceived as two substantial improvements over paper-based referrals. Most GPs felt that the SCI Gateway system was reasonably straightforward to use. Referral protocols and templates could be perceived as useful by some GPs while others considered them to be cumbersome at times.

**Conclusion:**

Our study suggests that the deployment and adoption of eReferral across the NHS in Scotland has been achieved by a combination of factors: (i) a policy context – including national mandatory targets for eReferral – which all NHS health-boards were bound to operationalise through their Local Delivery Plans and also (ii) the fact that primary care doctors considered that the overall benefits brought by the deployment of eReferral throughout the patient pathway significantly outweigh any potential disbenefits.

## Introduction

The volume of referrals from primary care doctors to secondary care specialists – for advice, a second clinical opinion and diagnosis, further investigations or clinical interventions – has been increasing steadily across health systems worldwide [1,2]. In the U.S., it was recently estimated that a third of patients are referred to a specialist service every year, and that specialist appointments represent over 50% of all outpatient visits [3]. In the U.K., a recent report by the King’s Fund suggested that there were in excess of 9 million elective referrals from primary care to secondary care in 2008, resulting in an annual spend of more than £15 billion for the National Health Service (NHS) in England [4]. In Scotland, the number of new outpatient appointments across the NHS was estimated to be around 1.4 million in 2009 [5]. By nature, access to specialist services in resource-constrained health services needs to be carefully – and ideally, optimally – managed [1,6,7]. Electronic referral (eReferral) can be described as the electronic transmission of patient data and clinical requests between health services providers. Previous studies have suggested that eReferral has the potential to introduce many benefits in health system systems, including: *cost-effectiveness and better utilisation of clinical and administrative resources, fast, secure and improved referral processes in primary care, standardisation and an increased completeness of patient referral data, improved communication and satisfaction with referral processes both in primary and secondary care, improved triage and management of referral requests in secondary care, decreased waiting times for outpatient appointments, a reduction in unnecessary specialist outpatient appointments, improved processes for urgent referrals, reduction in erroneous information, misinterpretation and referral mismanagement and overall improvements in quality and safety of care for patients*[8,9].

The National Health Service for Scotland (NHSScotland) has developed a national eReferral system now in widespread use across the health service. Recent figures estimated that in January 2011, the rate of electronic referrals across Scotland was 98.8% and that the electronic management of referrals was 81.4%^a^. In this study, we conducted 25 semi-structured interviews with a sample of General Practitioners (GPs, n=25) in order to identify the factors that have influenced the adoption and embedding of the national SCI (Scottish Care Information) Gateway system within the NHS in Scotland, in order to inform future implementations in this sphere.

## Background & related work

### Implementation of electronic referral systems

We performed a comprehensive scoping review on the deployment and evaluation of eReferral systems and found a limited number of studies reporting large-scale (i.e. national or regional) implementation experiences and evaluation of eReferral systems in the scientific literature.

Several countries have attempted – with varying degrees of success – to implement national eReferral systems, including England, Finland, Denmark, Norway, the Netherlands, New Zealand, Australia and the U.S. among others [8-16]. However, the deployment of these systems has often been slow and characterised by limited and localised uptake, or regional rather than nation-wide implementations.

A report by the Association of Chartered Certified Accountants (ACCA) and the Danish Centre for Health Telematics (MedCom) suggested that in 2004, 41% of healthcare referrals were performed electronically in Denmark and that this resulted in substantial cost savings and reductions in treatment delays [9].

In the NHS in England, the ‘Choose & Book’ service was designed to allow patients to choose, during the course of a primary care consultation, the place, date and time of outpatient appointments in hospital with the expectation that this would result in lower Do-Not-Attend (DNA) rates. The system has been in operation since 2004. However, a study of GPs’ and consultants’ perspectives on the system found no evidence of impact on DNA rates and mixed reactions regarding impacts on work processes [11]. Another study also suggested that patients did not experience the degree of choice that the ‘Choose and Book’ system was supposed to provide [17]. Surprisingly, we were unable to identify in the scientific literature a recent and comprehensive systematic evaluation of the ‘Choose & Book’ service in terms of usage patterns and impact on services and patient care.

In a questionnaire study with specialist consultants in a public hospital in the U.S, an iterative web-based electronic referral system between primary and secondary care significantly improved understanding of the reasons which motivated the referral, particularly in the case of surgical visits, and reduced the ratio of inappropriate referrals; however, this was a localised implementation across primary care providers and the San Francisco General hospital [13-15].

In New Zealand, eReferral is currently a key part of the National Health IT Plan designed to support improvements in the quality and delivery of health care services [16]. An Electronic Request Management System (ERMS) referral system was deployed in 2010 in the Canterbury health-board to handle referrals from primary care to other parts of the healthcare system, replacing letters and fax requests [18]. The referral requests handling is centralised before rerouting to the appropriate service. The system was designed collaboratively between primary care doctors and specialists. Key benefits of the system include: ease and convenience – with referral templates pre-population from the GP system –, guidance and individual feed-back and education to GPs. Interestingly, a recent report by the King’s Fund suggesting that more than 70% of GPs now used the system, despite being no financial incentives for doing so, thereby demonstrating that eReferral has been adopted in the health-board because it essentially streamlines the referral process [18].

A separate evaluation of 3 regional district health -oard eReferral implementations (Hutt Valley, Northland and Canterbury) have suggested a number of organisational and care management benefits, including faster, reliable referrals and improved referral processes from community to secondary services. A key recommendation for eReferral implementation was the sharing of referral protocols across health-boards [19].

### Electronic referral in NHSScotland

In Scotland, the Scottish Government has overall responsibility for the development and implementation of health and community care policies and the NHS in Scotland. NHSScotland [20] is organised into 14 regional NHS Boards, which oversee the provision of primary and secondary health-care services in each region, as well as being responsible for the implementation of national policies at the local level.

The multiple steps involved in the referral processes from primary care to specialist secondary care services were identified as important factors of accumulated delays in the care of patients. In 2008, eReferral management was identified as a critical enabler of the 18 weeks ‘Referral-To-Treatment’ target [21] and was defined as a key strategic eHealth policy priority under the NHS HEAT (Health, Efficiency, Access and Treatment) target programme [22]. All NHS Health Boards had the responsibility to ensure that primary care systems were able to send referrals via the national eReferral system (SCI Gateway) and that secondary care services were capable of receiving and triaging electronic referrals (eTriage). As a result, enormous progress has been made in making eReferral almost universal across the NHS in Scotland, reaching an estimated 98.8% of referrals as of January 2011^b^.

### The SCI gateway national eReferral system

SCI Gateway is a national electronic referral system developed by the Scottish Care Information (SCI) group, which is part of the Information Services of NHSScotland. SCI Gateway is designed to handle referrals directly from patient records held in GP systems and transfer these to secondary care systems, as well as handling shared care information and hospital discharge communications. Two accredited commercial GP information systems are currently used throughout primary care in NHSScotland, EMIS and Vision [23]. These systems interface with SCI Gateway so that relevant medical information can be automatically pulled from the electronic patient records to populate the electronic referral form.

**Figure 1:**
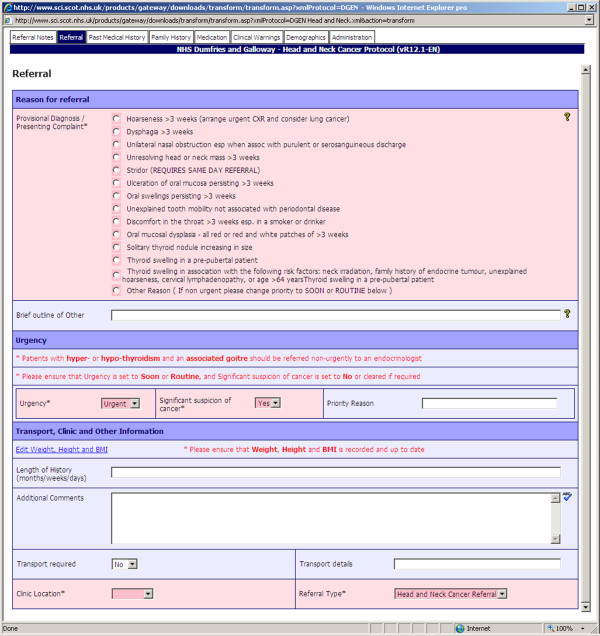
The SCI gateway eReferral system - user interface: Dumfries & Galloway ‘Head and Neck’ referral protocol.

The current stable version of the system is SCI Gateway Release 16.0 (10/07/12) [24]. The system is continuously maintained and a new iteration of the system is released once or twice per year. The SCI Gateway online resource provides comprehensive end-user and administrator support through documentation, newsletters, guidelines and release notes. Health-board referral templates are available publicly online through a comprehensive protocol library [25]. Figure 1 shows a screenshot of a referral protocol on the SCI Gateway user interface^c^.

The system uses standard internet technologies and an XML-format electronic document, based on standard referral recommendations issued by the Scottish Intercollegiate Guidelines Network (SIGN) [26,27]. Functionalities supported by the system include: clinical guidance, referral of clinical and demographic information, appointment booking and confirmation of appointment, and patient hospital discharge information updates. Each referral entered in the SCI Gateway system is mapped to one of 7 possible national defined statuses (i.e. appointment, test, procedure, cancellation etc.) [22].

## Methods

### Data collection

Our study design is described in details in Annex I (Additional file 1). Ethical approval for this study was obtained in February 2010 from the University of Glasgow College of Medicine, Veterinary and Life Sciences ethics committee. An invitation to participate in the study was sent to GP practices using a list compiled in April 2011 by the NHS Information Services Division [28]. We conducted 1 focus group with members of the Scottish eHealth electronic patient record programme (in August 2011) and one semi-structured interview with a member of the NHSScotland Scottish Care Information group responsible for the development of the national eReferral System, SCI Gateway (November 2012).

The primary care practitioners sample target size initially set for this study was between 20 to 25 participants which was successfully reached in January 2013 at which point no further GPs were recruited for this study. We conducted 25 semi-structured interviews with GPs and 1 focus group between February 2012 and January 2013. Interviews duration ranged from half-an-hour to above an hour, with a mean duration of approximately 40 minutes per interview. The interviews were semi-structured and open-ended in order to allow the interviewer or interviewee to elaborate on unanticipated and potentially valuable information with additional questions, and probe for further explanation [29]. The interviews aimed to collect GP views on information management processes in the patient surgical pathway in NHSScotland: *information about the GP practice itself, including information management practices and ICT use, the patient consultation and the referral process to hospital outpatient clinics, communication between GPs and hospitals from the point of referral to patient surgery, post-operative discharge information provided by the hospitals, issues identified in the patient surgical journey and areas for service improvement*[23,30-33]. The interview questions relevant to the referral processes have been included in Annex II (Additional file 2).

19 interviews were conducted over the phone and 6 face-to-face. Interviews were recorded with participant consent and transcribed verbatim. Fifteen of the GPs were male and ten female. Most of the interviewees had been practicing GPs for a considerable number of years, with a range of 1 to 35 years and a mean of approximately 16.5 years. Respondents were from 9 of the 14 territorial health-boards of Scotland (*GP1–GP6: from NHS Greater Glasgow and Clyde, GP7–GP11: NHS Ayrshire* & *Arran, GP12* & *GP13: NHS Dumfries* & *Galloway, GP14–GP16: NHS Fife, GP17: NHS Forth Valley, GP18: NHS Grampian, GP19–GP22: NHS Highlands, GP23: NHS Lanarkshire, GP24* & *GP25: NHS Lothian)*.

It was important to get respondents from as wide a sample of health-boards as possible as – although SCI Gateway is a unique eReferral system across all of the NHS in Scotland – the referral protocols themselves are health-boards’ specific [25].

### Data analysis

We analysed the data using an electronic health systems information management quality assessment framework for coding the transcripts of interviews [34]. The framework is derived from DeLone & McLean’s model of quality in information systems [35]. The framework comprises the following 6 dimensions: (i) eHealth information system quality, (ii) information quality, (iii) information usage, (iv) user satisfaction, (iv) individual impact and (vi) organisational impact. Through, iterative amalgamation of related codes, new distinct themes emerged which we grouped into three related thematic dyads presented in section “eReferral system evaluation”. The coding scheme was also used to derive descriptive statistics of the range of perspectives identified in our study sample described in the following section. For purposes of readability, we have also included essential excerpts of interviews to support our data analysis within the main article and – where appropriate – refer to relevant sections of the Annex III (Additional file 3) for additional quotes.

We then used Normalisation Process Theory (NPT) as a conceptual framework to interpret the factors which were identified as facilitating or hindering the work of GPs during the patient consultation. NPT is concerned with the social organisation of the work (implementation) of making practices routine elements of everyday life (embedding) and of sustaining embedded practices in their social contexts (integration) and was developed particularly in response to the evidence, which suggested that eHealth implementation, embedding and integration are difficult to achieve in practice [36-38].

NPT aims to explain the routine embedding of practices by reference to the role of four generative mechanisms: *coherence; cognitive participation; collective action and reflexive monitoring*. 

• **Coherence:** refers to the work of making a complex intervention hold together and cohere to its context, how people “make sense" or not of the new ways of working.

• **Cognitive participation:** is the work of engaging and legitimising a complex intervention, exploring whether participants buy into and/or sustain the intervention.

• **Collective action:** examines how innovations help or hinder professionals in performing various aspects of their work, issues of resource allocation, infrastructure and policy, how workload and training needs are affected and how the new practices affect confidence in the safety or security of new ways of working.

• **Reflexive monitoring:** is the work of understanding and evaluating a complex intervention in practice, and how individuals or groups come to decide whether the new ways of working are worth sustaining.

## Results: overall satisfaction & usage patterns

### GPs’ overall satisfaction with the SCI gateway system

We asked GPs to provide an overall opinion of the SCI Gateway eReferral system and then progressively refine their opinions in terms of perceived benefits and disbenefits of the various aspects and functionalities of the system.

Most GPs (16/25) were broadly satisfied with SCI Gateway, 6/25 expressed overall mixed feelings, 2/25 had an overall negative opinion of the system, and 1/25 had no overall opinion on SCI Gateway. These opinions are consistent with several other studies which have suggested that eReferral was perceived positively both by primary care health professionals and secondary care specialists as contributing to overall improvements in both patients’ referral management and care processes [8,13,15].

### SCI gateway system usage patterns

A very interesting aspect of the SCI Gateway usage pattern that became apparent during the course of our interviews – and one which was confirmed during a separate interview with the SCI system architect – is that many GPs do not actually fill in the referral form themselves. This is in marked difference to the usage pattern of practice information management systems, with which GPs interact directly during the patient consultation [23].

**Figure 2:**
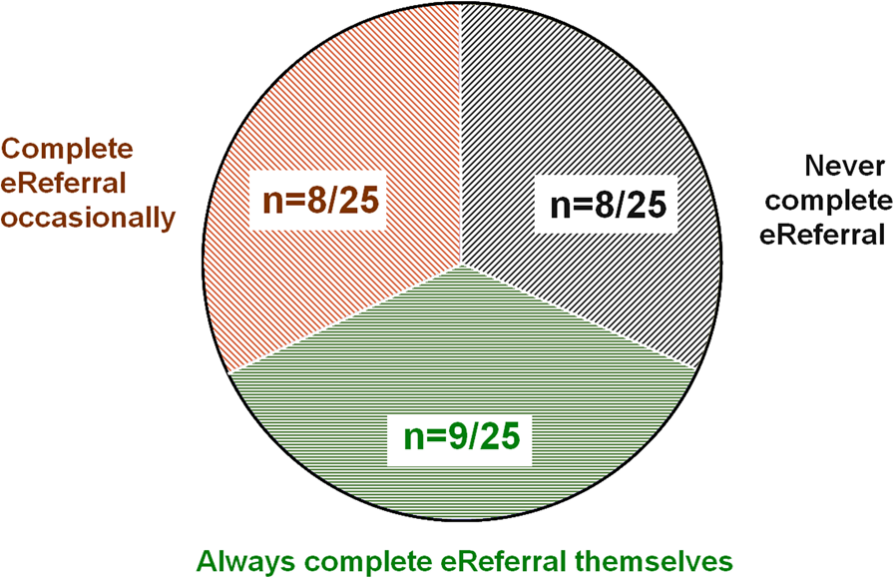
SCI Gateway usage pattern.

An overview of the SCI Gateway system usage pattern is presented in Figure 2. Nine out of twenty-five GPs declared that they completed and sent the electronic referral form themselves:

**• GP19:***“It’s good, yeah, yeah I quite like using it. We use to dictate our letters and our receptionist would put them through SCI but in the past year, I’ve started just doing the letters myself on the SCI Gateway”*

Eight out of twenty-five GPs declared that they completed the eReferral occasionally. GPs reported completing the electronic form when the referral was either urgent, if secretarial staff were not available for some reasons or if they thought the protocols were straight-forward enough for them to use:

**• GP6:***“It depends... I usually dictate them and then the typist will do them. If it’s an urgent one, depending on work and what time of the day it is, I will type it myself on SCI Gateway”.*

**• GP9:***“I do myself for some referrals, yes, ones which we use to always make on paper light, open endoscopy, physiotherapy, audiology, open access... sorry: rapid access, chest pain. These are ones I would do myself... [...] For the ones that don’t have much narrative in them they’re very good. And I will happily do the tick box referrals, but if I have to do a big dictation, then I would prefer the secretary to type it”.*

A further eight out of twenty-five GPs declared that they recorded the referral on a dictaphone and that secretarial staff then completed the electronic referral on SCI Gateway. The referral was then usually “parked” by secretarial staff so that it could later be reviewed by the GPs who - if satisfied that the referral was clinically correct - then sent on the referral themselves.

**• GP3:***“... All done electronically. We dictate it into a machine very similar, in fact almost identical to that...* (i.e. the interviewer’s) *digital recorders and then it just, it downloads onto the computer and the typist, we have a typist who puts them all on the SCI referral system and then we check and send them. That’s it. So it’s all done electronically”.*

There was an interesting variation to the above practice worth highlighting here, as it has important implications both in terms of work processes and the potential costs of eReferral. One GP declared that the doctors in her practice simply dictated the referrals and that the referral was then typed and sent by an experienced secretary.

**• GP6:***“...I don’t review them actually because I’m not there until the following Wednesday”*

**Interviewer:** “so who reviews them?”

**GP6:***“in [practice name] no-one does, the typist has been there for 20 years and every one’s quite happy for her to send them”*

These results on the eReferral completion and submission pattern is in sharp contrast to another study which reported that eReferral were generally submitted directly by the treating primary care practitioners [13].

In our study, it would appear that the current mixed usage pattern can in part be explained as a *legacy* of paper-based work processes. Before the eReferral system was implemented across the NHS in Scotland, GPs used to record their referral letters orally through a dictaphone. The letters were later typed and sent to the relevant services by the practice administrative support staff. Our results suggest that in many cases, this process has now simply been transposed to the eReferral. The pattern is particularly evident among GPs with the longest years of practice, suggesting that this group was the most reticent to use the eReferral system directly.

Although this was beyond the scope of our study, it would be useful to measure in future the cost implications of this *“hybrid”* adoption of the eReferral system to assert which usage pattern is generally the most cost-effective to the practice. While it may seem initially sensible from the GPs’ perspectives to delegate more time-consuming administrative tasks to secretarial staff who command lower labour costs, other studies have also suggested that delaying the adaptation of work-practices to new IT systems could also result in the economic benefits of the new technology not being realised to their full potential [39]*(see also Annex III.1.1 (Additional file*3*) for the description of an example of an ad-hoc ‘work-around’ for eReferral management implemented in a specific GP practice)*.

## eReferral system evaluation

Following on from these broad and initial perspectives on overall satisfaction and usage patterns, we asked GPs to try to further elaborate on more specific aspects of the SCI Gateway system which they perceived as either facilitating or hindering their work in the course of the patient referral.

The result of the qualitative analysis of GP’s responses are here presented in the following 3 thematic dyads, using the eHealth system quality framework derived from DeLone & McLean’s model of information systems’ quality [34,35]: 

(i) information system and information quality (in section “SCI gateway system & information quality”),

(ii) information usage and user satisfaction (section “Information usage & user satisfaction”),

(iii) individual and organisational impact (section “Individual & organisational impact”).

### SCI gateway system & information quality

#### Perceived benefits of SCI gateway system

Respondents described the following beneficial features of SCI Gateway: 

- the SCI Gateway system provides adequate support for automated data entry: (n = 12 GPs)

- it allows providing additional information during the referral if needed: (n = 6 GPs)

- it provides adequate work-flow support during the patient consultation: (n = 2 GPs)

• Support for automatic data entry:

SCI Gateway interoperates with the 2 accredited GP information management systems used throughout the NHS in Scotland [23] and can pull data automatically from the electronic patient record whenever relevant to complete the referral, *(see also Annex III.1.2 (Additional file*3*))*.

**• GP8:***“...I think one important advantage is that it automatically populates the referral with past... with important past information, that we don’t need to edit and complete that, it does it automatically which is very helpful”.*

**• GP25:***“ The biggest benefit in the SCI system is the... number 1 is obviously you’re not having to... information is copied automatically from the GP system and that makes quite a big difference... not having to put in somebody’s past medical history is very useful and it does that and it pulls that over automatically and not having to put in their drug and medication history again is very useful... and that’s pulled over accurately”*

• Support for providing additional clinical information:

As we have suggested earlier in section “The SCI gateway national eReferral system”, the SCI Gateway referrals are usually protocol-driven, which entails providing pre-determined and relevant sets of information to allow appropriate screening and triage in secondary care. In addition, the system permits additional discretionary information in the form of comment boxes which allow the addition of clinical comments (see also Figure 1). Several GPs felt that this provided added flexibility for sharing relevant clinical information with secondary care practitioners, *(see also Annex III.1.3 (Additional file *3*))*.

**• GP23:***“...you can cut and paste the consultation itself then, with a bit of tweaking then, that tends to, I think, give them a good background as to the reason for referral”.*

• Support during the patient consultation work-flow:

Two GPs felt that the SCI Gateway system could be used within the consultation:

**• GP10:***“...we can see the patient, see the patient and sometimes your... depending on if it’s a protocol that I’m happy using on SCI, I can have the referral done with the patient in the room, press a button and know it’s gone. You know, it may not be seen for a while, but I know that I’ve done it, and it’s sent instantly which I think is very good”.*

#### Perceived Dis-benefits of SCI gateway system

GPs also reported some perceived limitations with the SCI Gateway system, including the following aspects: 

- SCI Gateway can be administratively cumbersome and does not always adequately support the patient consultation workflow: (n = 9)

- the system is too slow: (n = 6)

- information presentation and visualisation and system status and feed-back are not always adequate: (n = 5)

- the system occasionally breaks down or can lead to data loss during referral transfer: (n = 4)

• Administratively cumbersome:

A previous study on the coordination of electronic referrals between primary care and secondary care professionals and patients found that while protocol-driven referrals could be perceived as constraining by primary care professionals, secondary care consultants were of the opinion that template-based referrals improved the overall quantity and quality of information they received from their primary care colleagues [40]. It is therefore perhaps unsurprising that while an eReferral protocol-driven system will promote the standardisation and reduce variations of referrals, it will also entail additional clinical and administrative tasks for the referring GPs and potentially introduce some degree of frustration among end-users. This aspect of eReferral has also been highlighted in a recent report on eReferral commissioned by the Agency for Healthcare Research and Quality (AHRQ) [8].

*(see also Annex III.1.4 (Additional file *3*))*.

(when asked about the SCI protocol-driven referrals):

**• GP6:***“...What’s frustrating is when they have um... what do you call them... mandatory boxes that you can’t submit it unless you give the information but if you don’t... sometimes you don’t have it all and then that’s a bit of a... you know... a bit of an issue”.*

**• GP23:***“...I think it can be a good thing for the people that you’re referring to because they get the information that they request. Sometimes it’s difficult to remember the smaller points that every body wants to be done. And the patient... they will have left the room and you’ve not asked or you not arranged for a particular little thing to be done that was on the referral criteria. Because you’ve got to wait till you’re going through it to actually tick the relevant boxes”.*

• Issues of system performance:

Several GPs complained that the system could at time be frustratingly slow, although it appeared unclear whether this was a performance issue specific to SCI Gateway, or related to the level of internet connectivity at individual practices, *(see also Annex III.1.5 (Additional file *3*))*.

• Information presentation & system status:

The SCI Gateway system allows referrals to be “parked” before sending off. This is presumably to allow for the results of additional investigations to be added later on before the actual referral request is made, or for secretarial staff to complete the referral with a typed clinical letter before sending off. Two GPs mentioned that this feature could cause some confusion at times. Some GPs who had completed a referral had thought that it had been sent when it was in fact still pending. This type of issue would probably be effectively addressed if GPs simply received additional training with their IT systems [23]. It also emerged during the interviews that it was common for practice secretarial staff to monitor the status of referrals on the system and simply remind GPs to complete the referrals whenever some had been left pending for some time, *(see also Annex III.1.6 (Additional file *3*))*.

2 GPs complained about what they perceived as inadequate information visualisation and presentation:

**• GP1:***“...like most IT things it is overly detailed and you can’t see you... It makes it hard to see what you want to see. You get flooded with lots of information and it’s actually very difficult to sift out the knowledge that sits within that information”.*

**• GP7:***“...Well there seems to be an awful lot of unnecessary information on them that could either be sent without being visible to make the templates simpler or just doesn’t need to be sent at all I suspect”.*

• Data loss and system breakdown:

Overall, the SCI Gateway system appeared generally stable and reliable system. However, as with any IT system, occasional system failures are inevitable, even if infrequent:

**• GP8:***“...we have encountered at the recipients’ end... we have sent a referral and I don’t know of any that have been lost, but some have been incomplete upon receipt: they appeared to leave us intact, but attachments and so on, things are sometimes lost... in the sending process. but I think these are glitches that have probably been addressed and solved”*

**• GP21:***“...very occasionally we had occasional instances were it’s not worked well, were a referral is a... we thought it’s been sent but it hasn’t or it hasn’t been received so but that’s maybe once a year”.*

Although in the latter point, the data loss attributed to the eReferral system could also have been the result of issues with the GPs handling of the electronic referral, as has been highlighted previously (e.g. confusion about eReferral statuses).

### Information usage & user satisfaction

#### Perceived benefits

Most GPs (16/25) reported benefits in terms of information usage and user satisfaction, including: 

- the SCI Gateway system is perceived as useful and/or has good usability: (n = 11 GPs)

- the immediate transfer of the referral request is perceived as a key benefit: (n = 9 GPs)

- clinical advice and referral guidance functionalities were perceived as useful: (n = 5 GPs)

• Usefulness & usability of SCI gateway system:

GPs were reasonably satisfied with the usefulness of the SCI Gateway system and the usability of the user interface. We have previously reported that GPs can be pragmatic – yet at times reticent – users of IT systems [23]. In addition, perceived ease of use and an intuitive user interface have been identified as key factors promoting the successful adoption of eReferral [8].

**• GP13:***“ I think to be fair, the referral system, the user interface were quite OK, quite good... huh, the guidelines links didn’t always... were not always useful but there... that was not a big issue because I know where to look for [...] I know where to get information I am looking for, so [...] yeah, I think all in all, it’s a good system”.*

**• GP24:***“yeah, it’s quite intuitive, it’s quite good, it’s a bit on the slow side but I quite... I find it quite OK”*

• Immediate transfer of the referral request:

The immediate transfer of the electronic referral was often perceived as a key improvement on previous paper-based referrals by GPs, as well as being an element of the system which seemed to *‘lift a weight off their minds’*. Previous studies have also highlighted how healthcare proffesionals have identified immediate information transfer between services as a key benefit of electronic clinical communication [16,41].

*(see also Annex III.2.1 (Additional file *3*))*.

**• GP17:***“The advantages: you know it’s gone because you get a... you get a confirmation that it’s been received. Whereas if I send a letter, sometimes it gets lost in the hospital. You know it can go immediately, you know it’s much quicker than posting [...]”*

**• GP22:***“... Yes it’s excellent, I mean if I’m putting someone through at 8 o’clock at night you know that this is going to be at least looked at first thing the next morning... and it’s fabulous”*

• Clinical advice/referral guidance functionalities:

Several studies have previously suggested that GPs often have insufficient knowledge to best use clinical advice and decision support functionalities provided by their computing systems [42,43]. The fact that SCI Gateway made explicit clinical advices via the referral protocol interface was perceived as helpful by several GPs:

**• GP2:***“... Some of the stuff like might have specific form [...] so it kind of helps focus you to what you should be doing on some of these forms, it’s just really good”.*

**• GP15:***“Gateway is quite good at... obviously it won’t let you send it if there’s something missing from the box... it makes sure the right level of information gets, gets to the doctors outside...it depends on the specialty but it might, you know... it won’t let you refer if you missed certain boxes and say ‘have you done this? have you considered that?’ so that can be helpful yeah...”*

#### Perceived Dis-benefits

7/25 GPs reported limitations with the usability of SCI Gateway such as referral protocols being rigid or check boxes duplicating information already contained in adjoining comments or an enclosed clinical letter. Naturally, these aspects of the SCI Gateway system are inherently a consequence of the constraints imposed by a standard protocol-driven interface, which we have discussed in the previous section “SCI gateway system & information quality”. From the secondary care consultants’ perspectives, a structured referral protocol ensures that the information that they need is provided regardless of whether it has also been included or not in an adjoining unstructured clinical letter, as well as knowing exactly where to find it [14,44]. This aspect was not always adequately appreciated by the referring GPs *(see also Annex III.2.2 (Additional file *3*))*.

### Individual & organisational impact

#### Perceived positive impacts

Perhaps unsurprisingly for a electronic referral system, which by definition provides information to be used by a third-party, the greatest benefits of the SCI Gateway system were expressed in terms of individual and organisational impacts. 

- the greatest individual impact to work practice mentioned by GPs’ was an increased use of standard and guidelines during the referral, (n = 16)

- from an organisational impact, several GPs felt that the electronic referral improved organisational work-processes and performance and patient management across the health services (n = 8)

- eReferral improved the integration of heterogeneous electronic information systems and information sharing across the health services (n = 5)

• Improved use of standard protocols and guidelines:

Although we have already covered aspects of the protocol-driven referrals both in terms of system quality and user satisfaction, an additional element of standard clinical referrals was the potential contribution to an increased awareness, knowledge and use of referral protocols and guidelines. The potential impact of eReferral for primary care education and knowledge transfer has also been highlighted in a recent report on eReferral commissioned by the AHRQ [8].

*(see also Annex III.3.1 (Additional file *3*))*.

**• GP13:***“....there are often referral protocols where you have to kind of... tick boxes if you want to use a particular service... huh... and it often guides you to use a particular service where you can direct your referral to.. humhhh... and again I think those systems are working quite well [...]*”

(about the potential variations in the protocols:)

“I think there were lots of variations in different specialties so for instance, the place where I work, they had very good protocols for gastroenterology, but other specialties didn’t have such good protocols”

**• GP17:***“...this SCI Gateway system that we use it links to guidance... So if we’re making the referral it will have guidance about who’s appropriate to refer and who’s not appropriate to refer. And finally it can prompt for things, so it can prompt you to you know check a blood pressure or it can prompt you to, you know, put information about disabilities or past medical history or things that you might forget”*

• Improved organisational work processes and performance:

Several GPs suggested that eReferral introduced a number of organisational and performance benefits, including improved follow-up and tracking of referrals as well as improving processes for emergency referrals, *(see also Annex III.3.2 (Additional file *3*))*:

**• GP12:***“...Well if we suspect cancer, there is box on our SCI referral that you tick. You know: ‘suspect cancer’, that is, it’s taken as an urgent referral and also the ‘managed clinical network for cancer’: they follow those guys up and make sure that things are, you know, that things are happening and everything. I mean... they have worked hard to work at their... reduce bottle necks where there have been, like you know, investigations or something where there was delays and stuff and things... they’ve tried hard to try and make sure that those, particularly for the cancer patients, are sorted out so those patients are investigated and sorted out as soon as”.*

• Improved information sharing/systems integration accross the health services:

The fact that SCI Gateway is able to provide feed-back to GPs on referral statuses and can link-up with the other IT systems used in the practice was perceived as positive [23], *(see also Annex III.3.3 (Additional file *3*))*.

**• GP9:***“...I quite like the various features of it; that you can see that they’ve looked at your referral and they’ve acted upon it, so, yes I’m fairly happy with it”.*

#### Perceived Dis-benefits

Some GPs (10/25) GPs expressed some concerns about effects on individual or organisational work processes, with several feeling that the electronic referral was more complex or time consuming than previous paper-based referrals (n = 5). From an organisational impact perspective, several GPs felt that a lack of coordination across the health system could result in GPs incurring work overheads to suit third parties work processes or the referral process not being entirely transparent or coherent to GPs (n = 5), *(see also Annex III.3.4 (Additional file *3*))*.

#### Other organisational & socio-technical factors

**eReferral is designed to suit the information needs of the recipient of the referrals**: Perhaps unsurprisingly due to the very nature of a referral, several GPs (n = 7 GPs) felt that the SCI Gateway system was essentially geared towards the information needs and work processes of third parties within the NHS. This aspect of referral templates being perceived by primary care practitioners as being essentially designed for the convenience of secondary care consultants has been observed in other studies [8,40]. Nevertheless, it seemed clear in our study that GPs understood the necessity for this. Although this implied additional overheads, the rational for this seemed often – and at times with some degree of reticence - accepted by the GPs as being both necessary as well as overall beneficial to patient care and management, *(see also Annex III.3.5 (Additional file *3*))*.

**• GP3:***“Some of it is O.K. I mean, I think – like you know... – the colorectal one’s a pain. But it actually is not too bad, you know... It’s really for them to be able to detect the urgent ones compared to the non-urgent: that’s the idea and that’s fair enough! I’ve no problems with that”.*

**• GP8:***“Well from our end, from our point of view... yes there are advantages in doing it, overall yes, although I think it’s probably more helpful for the recipient of the referral”*

• Lack of feed-back on referrals:

A common grievance mentioned by GPs was related to the lack of feed-back on the progress of the referrals once they have been sent(also n = 16 GPs). This aspect is not directly due to the SCI Gateway system itself but is rather a consequence of clinical processes in secondary care. Once a referral has been received and triaged to the appropriate service at the hospital, secretarial staff at the service will then communicate directly with the patient. GPs are no longer kept in the loop up until they subsequently receive a letter from the outpatient services summarising the clinical findings of the consultant – or a Did-Not-Attend notification if the patient did not present for his appointment. Although GPs generally did not want to be inundated with e-mail notifications about patient appointments, they would have liked to be able to check a referral status if and when patients came to enquire about the progress of a referral.

In fact, SCI Gateway includes such a functionality, allowing explicit feed-back to be provided to GPs. They can check on the system whether a referral request has been received and read, so the issues here were multi-fold.

Some GPs did not seem to know about this functionality, others did but argued that knowing that a referral had been read did not give any indication as to whether it had actually been actioned and how long it would be before a patient would be seen at the outpatient clinic. The SCI Gateway system developer we interviewed was aware of this issue and again emphasised that this was essentially a clinical process issue in secondary care and not a technical issue. The system itself has the means to convey this information to GPs but hospitals do not generally provide it to the system, *(see also Annex III.3.6 (Additional file *3*))*.

**• GP4:***“"[...] I don’t think you would need to receive a notification elect... through an e-mail or anything because I think you would just get a lot of them and you would end-up probably not... but it would be useful to be able to go in and check so if a patient says ‘I’ve still not received the letter’, it would be useful to be able to go in the system and say well you’re in this stage so it won’t be that long [...] they do sometimes say: ‘I was referred months ago but I haven’t hear anything’...”*

**Interviewer:**...so what happens then? What do you do?

*“...you have to just phone up the secretary”* (at the hospital)

• Lack of coordination across the health services & lack of work practice coherence:

These aspects are mainly related to issues around the coordination of referral pathways and the onward processing of referrals across the health services. In particular, local NHS health-boards have the responsibility to develop referral protocols and map referral services and this is naturally an iterative development, by which priority services and protocols are implemented first while other services have no specific protocols and on occasions not mapped or available for referral via the SCI Gateway, *(see also Annex III.3.7 (Additional file *3*))*.

**• GP2:***“The only confusing thing about is that some things, and more and more is on SCI Gateway now, but there’s still some services that are not on SCI Gateway. It’s sometimes difficult to remember what’s on SCI Gateway and what still needs some other specific form on whatever. [...] Sometimes you have to look on it to see, you know, how do you refer these things..... [...] It can be quite difficult to find out. So that sort of thing”*

## Interpretation & discussion

Using the 4 NPT constructs, we now review and interpret the findings of our study in turn:

• Coherence:

Coherence refers to the “sense-making” work undertaken when a new e-health service is implemented: to determine whether users see it as differing from existing practice, have a shared view of its purpose, understand how it will affect them personally and grasp its potential benefits [45].

It is clear that considerable effort was put into policy building and dissemination of information both locally and nationally in relation to the national eReferral system. At the turn of the millennium, the Scottish Government set an ambitious three year national programme to implement widespread electronic clinical communication between primary and secondary care by 2003: the Electronic Clinical Communications Implementation (ECCI) programme. The implementation of ECCI was a considerable challenge due to a multitude of factors, including: *the wide variability of local IT infrastructures across health-boards, variations in the technologies used, available personal and resources, training, organisational and stakeholders’ support and variations in the perceived benefits of the implementations*[46,47]. In 2005, the ‘Delivering for Health’ programme required that NHS boards develop three year implementation plans over five quality improvement priorities, including improving referral and diagnostic pathways. eReferral to a central point was a key element of the strategy [7,48]. In 2008, eReferral was defined as a key strategic eHealth policy priority under the NHS HEAT target programme [22].

The key lesson here is that the success of the implementation of a national eReferral system in the NHS in Scotland did not happen overnight. Several national strategic plans were necessary to provide the IT infrastructure and resources necessary for successful deployment of the national eReferral system. Several of these strategic plans did not meet their initial targets. The successful adoption of eReferral in Scotland – both in terms of volume and overall satisfaction of end-users, as is suggested by our study – therefore needs to be seen as the result of the provision of a national integrated infrastructure for eReferral through the SCI Gateway system combined with *a sustained effort* to engage with key stakeholders and allow changes in practices, culture and IT use within NHSScotland to take place over a decade and a half.

• Cognitive participation:

Cognitive participation focuses upon the work undertaken to engage with potential users and get them to “buy into" a new e-health system [45]. The work of relating and engaging with users is central to the successful implementation of any new technology.

A protocol-driven eReferral system such as SCI Gateway requires GPs to ensure that all relevant mandatory information has been provided to the system before allowing the referral request to be sent off. GPs understood the need for protocol-driven referrals, while at the same time suggesting variations in the implementation and quality of the protocols. Some GPs described certain protocols as good, useful and contributing to their understanding of the information required for certain referrals. Others found them at times cumbersome and frustrating. The ability for GPs to provide additional information during the referral in the form of attached documents and clinical letters gives them a degree of flexibility in terms of the information that they provide to secondary care services. On the other hand, if template-based protocols are ignored only for the patient clinical information to be provided in the form of an unstructured clinical letter, this also runs the risk of defeating the purpose of the standard referral document in the first place.

From the perspective of the secondary care practitioners who have designed the referral protocols, this is to ensure that all necessary steps have been taken in primary care before the clinical case for the forward referral to a specialist service has clearly been established. Previous studies have suggested that a surprisingly high number of secondary consultants often do not understand why a particular patient has been referred to their service based on the information that has been provided in the referral letter or that the information provided is inadequate to make a proper patient management decision, resulting in delays, the duplication of investigations or underuse or overuse of services [3,14,44].

• Collective action:

The emphasis of collective action involves the work performed by individuals, groups of professionals or organisations in operationalising a new technology in practice and socio-technical issues, such as how e-health systems affected the everyday work of individuals and organizational structures [45].

Many GPs accepted the need for standardised protocol-driven referrals, however, it appeared unclear how much involvement primary care practitioners had in developing the protocols or if these were essentially designed by secondary care professionals with minimal or no consultation with their primary care counterparts. Although there will naturally be legitimate variations in the information needs across different clinical specialties, a concerted effort across health-boards to improve the coherence and standardisation of referral protocols across specialties is also essential to maximise the potential benefits of the standardisation of referrals across NHSScotland and to keep the process as simple as possible for primary care professionals.

Such an approach simplifies the work of both primary and secondary care practitioners as it reduces the need to navigate diverse and complex referral protocols for the former while reducing the effort required from the latter when handling heterogeneous referral documents of varying quality. The need for greater coordination across specialties and the development of mutually agreed referral protocols between primary and secondary care practitioners has been recommended in several recent studies [14,19,40,44,49]. Such a collaborative approach between primary and secondary care practitioners has for example been reported by Dennison et al., albeit at a localised/pilot implementation level [50]. A recent report by the King’s Fund on initiatives to improve health and social care integration in Canterbury, New-Zealand, describes the development of *‘HealthPathways’*, which are local referral pathway agreements between primary and secondary care in the health-board [18]. They were developed as secondary specialists eventually realised that a key strategy to effectively manage demand for specialist services was to *engage* directly with primary care doctors in order to identify joint solutions to address the critical issues of inappropriate referrals and service bottlenecks. More than 480 *HealthPathways* have been developed, combining electronic referrals (ERMS) with online referral guidance for GPs and information for patients. Some of the impact of the initiative have included a greater number of cases managed and treated by GPs, an increase access to diagnostic testing (e.g. spirometry) in primary care and more appropriate referrals in secondary care.

The lack of feed-back from secondary care on referral progress was a common complaint among GPs and has been identified in other recent studies of eReferral [8,40,49]. A system implementing electronic notifications to the referring primary care providers has been described by Kim et al. [13]. However, the system had been implemented in a single hospital and therefore the feasibility of implementing this feature on a regional or national scale can not be generalised. The practical difficulties which would be entailed by a national roll-out of eReferral notifications to primary care providers – both in terms of the required additional administrative tasks handled in secondary care as well as the potential impact on primary care workflows – should therefore not be underestimated.

• Reflexive monitoring:

Reflexive monitoring deals with the evaluation and monitoring of eHealth implementations and how these are used to influence utilisation and future implementations [45].

For the SCI system, much of the reflexive monitoring around eReferral seems to take place during the iterative development life-cycle of the system. Usability and technical issues identified by end-users of the system can be reported back to the individual health-boards eHealth leads or the SCI development team [51]. The SCI development team can then address these in subsequent annual releases and end-users’ communications, as we have highlighted previously in section “The SCI gateway national eReferral system”. In addition, health-boards have made their referral protocols publicly available online through a comprehensive protocol library, hence allowing convenient knowledge reuse and transfer across health-boards, transparent scrutiny of referral protocols as well as providing an invaluable implementation reference resource for all developers of eReferral protocols and systems world-wide.

### Study strengths & limitations

The main strengths of this study lie in fact that we have used a robust analytical framework, combining information system quality and implementation process theory (NPT) in order to analyse primary care perspectives on the national eReferral system. Another strength is that with interviewees from 9 of the 14 territorial health-boards, the sample is geographically reasonably representative of NHSScotland. There are also a number of limitations to our study design which need to be considered. The target sample of 20 to 25 primary care respondents was somewhat arbitrary and determined by our capacity to subsequently analyse the data within the duration of this research project. The second limitation is that participants who have volunteered to participate in this study may have had potentially a greater interest in research or the topic of the study (i.e. respondent bias). These limitations means that while our results present a useful snapshot of the perspectives of a sample of end-users of eReferral in NHSScotland, these may not necessarily be generalisable to the whole population of GPs in Scotland. We are hoping to complement this study in future with a larger online study of stakeholders’ perspectives on eReferral.

## Conclusion

The majority of GPs interviewed felt that eReferral substantially streamlined communication processes, with the immediate transfer of referral documents and the availability of an electronic audit trail perceived as two substantial improvements over paper-based referrals. Most GPs felt that the SCI Gateway system was reasonably straightforward to use. Referral protocols and templates could be perceived as useful by some GPs while others considered them to be cumbersome at times. Our study suggests that the deployment and adoption of eReferral across the NHS in Scotland has been achieved because primary care doctors considered that the overall benefits brought by the deployment of eReferral throughout the patient pathway significantly outweigh any potential disbenefits.

## Endnotes

^a^ eReferral, HEAT Target, E7 http://www.ehealth.scot.nhs.uk/?page_id=482

^b^ eReferral, HEAT Target, E7 http://www.ehealth.scot.nhs.uk/?page_id=482

^c^ Dumfries & Galloway ‘Head and Neck’ referral protocol: DGEN Head and Neck.xml http://www.sci.scot.nhs.uk/products/gateway/downloads/transform/transform.asp?xmlProtocol=DGEN\ %20Head\%20and\%20Neck.xml&action=transform

## Abbreviations

AHRQ: Agency for healthcare research and quality; CDSS: Computer clinical decision support system; ECCI: Electronic clinical communications implementation programme; ENT: Ear-Nose-Throat; eReferral: electronic referral; ERMS: Electronic Request Management System (Canterbury, New-Zealand *HealthPathways*); GP: General practitioner; ICT: Information & communication technology; NHSScotland: National Health Service for Scotland; NPT: Normalisation process theory; SCI: Scottish Care Information group; SCI Gateway: The national eReferral system developed by the Nantional Services Scotland SCI group; SIGN: Scottish Intercollegiate Guidelines Network.

## Competing interests

The authors declare that there are no conflicts of interests.

## Authors’ contributions

M-MB and FSM conceptualized the project. M-MB conducted all visits on the site and conducted the semi-structured interviews, data collection and analysis, performed the literature and internet searches and drafted the original submission of this manuscript. FSM critically reviewed and revised subsequent versions of the manuscript. All authors read and approved the final manuscript.

## Pre-publication history

The pre-publication history for this paper can be accessed here:

http://www.biomedcentral.com/1472-6947/14/30/prepub

## Supplementary Material

Additional file 1 ANNEX I – A qualitative evaluation of general practitioners’ views on protocol-driven eReferral. Click here for file

Additional file 2 ANNEX II – Semi-structured questionaire Part II - Referrals. Click here for file

Additional file 3 ANNEX III – Additional quotations from the study participants. Click here for file
