# Supplementary material for: A qualitative evaluation of general practitioners’ views on protocol-driven eReferral in Scotland
Source: BMC Med Inform Decis Mak. 2014 Apr 9;14:30. doi: 10.1186/1472-6947-14-30 (PMC4101875; doi:10.1186/1472-6947-14-30)
Supplement: Additional file 1 — ANNEX I – A qualitative evaluation of general practitioners’ views on protocol-driven eReferral. [file 1472-6947-14-30-S1.pdf]

ANNEX I - A Qualitative Evaluation of General Practitioners' views on Protocol-Driven eReferral in Scotland

Qualitative RATS checklist

Matt-Mouley Bouamrane & Frances S. Mair

Research questions:

- What are GPs' views on their electronic referral system (this study)?
- What are the referral processes used in primary care in NHS Scotland in the course of the patient elective surgical pathway (this study)?
- What are the information management processes and systems in place in the course of the patient elective surgical pathway? What are their benefits and dis-benefits? *(broader Chief Scientist Office, CSO project study)*
- How can integrated care pathways in elective surgery improve the delivery of care for patients scheduled for elective surgery? *(broader CSO project study)*

Qualitative method:

- semi-structured interviews with GPs (n=25) and 1 focus group
- 1 focus group with members of the Scottish Electronic Patient Record programme
- one interview with a senior architect of the Scottish Care Information national SCI Gateway eReferral System
- *interviews were semi-structured and open-ended in order to allow the interviewer or interviewee to elaborate on unanticipated and potentially valuable information with additional questions and probes for further explanation*

Sampling / Recruitment / Data Collection:

- Ethical approval for this study was obtained in February 2010 from the University of Glasgow College of Medicine, Veterinary and Life Sciences ethics committee
- volunteer participants, invited by letter, across all 14 territorial health-boards of NHS Scotland using a list of practices compiled by Information Services Division, ISD Scotland
- recruitment target of n=25 GPs, based on research capacity within this project
- all participants provided informed consent
- a potential limitation of the study is respondent bias, as only GPs who responded to the invitation to participate in the study were subsequently interviewed. We have no additional information on non-respondents
- interviews were conducted either over the phone (n=19) or face to face (n=6)
- interviews with GPs were conducted between February 2012 and January 2013
- interviews were recorded with the consent of participants and transcribed verbatim
- interview questions on referral are listed in annex I
- data collection ended when recruitment target was met and project ended

---

Data analysis:

- DeLone & McLean's model of quality in information systems was used for data coding
- coding scheme used for descriptive statistics of responses in our study sample
- Normalisation Process Theory (NPT) was used to interpret study findings.
- NPT is a robust theoretical framework, specifically appropriate for the study of implementation processes in complex and / or technological interventions
- both benefits and dis-benefits have been identified, reviewed and discussed in the study
- quotes were chosen to illustrate the findings under DeLone & McLean's model of quality in information systems
- additional quotes have been provided in Annex III
- themes were discussed and reviewed by 2 researchers, one being a computer-scientist / health-informatician (M-M.B) and the other a GP and Professor of Primary care research (F.M.), bringing a balanced interpretation of the study results thereof, taking into consideration the perspectives of both implementers and end-users of eReferral
- strengths of the study include: coding framework developed from a widely used model in information system science, complemented by a robust theoretical framework, NPT. In addition, the study is broadly representative of the geographic diversity of NHSScotland, with participants from 9 of the 14 territorial health-boards in Scotland.
- limitations of the study include: small sample of respondents, with thus the potential for respondents' bias. Size of study sample limited by the project research capacity. No information on non-responders is available.
