# Supplementary material for: A qualitative evaluation of general practitioners’ views on protocol-driven eReferral in Scotland
Source: BMC Med Inform Decis Mak. 2014 Apr 9;14:30. doi: 10.1186/1472-6947-14-30 (PMC4101875; doi:10.1186/1472-6947-14-30)
Supplement: Additional file 2 — ANNEX II – Semi-structured questionaire Part II - Referrals. [file 1472-6947-14-30-S2.pdf]

ANNEX II - Semi-Structured Questionaire
Part II - Referrals

A Qualitative Evaluation of General Practitioners' views on Protocol-Driven eReferral in Scotland

Matt-Mouley Bouamrane & Frances S. Mair

Part II - Referrals:

- How long does a consultation with a patient last?
- When do you make the decision for a referral to secondary care?
- How often would you make a referral to secondary care?
- What criteria do you base your decision on? Would you base your decisions on tests, observations, patient narrative?
- What protocols do you use for diagnosis tests?
- What is the patient influence on the decision for a referral?
- How do you communicate to the patient a decision for a referral?
- How do you feel that patients react?
- Do you provide additional information at the stage of referral and in what form?
- Do Patients seek ask additional information at this stage?
- How do you communicate your decision for referral to the consultant?
- What items of information do you include in a referral letter?
- Do you use referral pathways / guidelines?
- How do you keep up to date with referral pathways / guidelines?
- How often would you say this referral subsequently lead to surgery?
- How do you complete the referral to secondary care?
- Which hospitals to you make your referrals to? Why?
- Do you select a specific specialty or a named specialist?

- How do the hospitals / consultants keep you up to date of patient progress, (e.g. appointments, outcomes of medical appointment, decision to operate, surgical-pre assessment, discharge, etc.)
- How involved are you at this stage of the process and would you want to have an additional input in the process?
- What are the modalities of communication with secondary care that you use?
- What further information / interventions do secondary care staff ask of you? What is your opinion of these requests?
