# Supplementary material for: A qualitative evaluation of general practitioners’ views on protocol-driven eReferral in Scotland
Source: BMC Med Inform Decis Mak. 2014 Apr 9;14:30. doi: 10.1186/1472-6947-14-30 (PMC4101875; doi:10.1186/1472-6947-14-30)
Supplement: Additional file 3 — ANNEX III – Additional quotations from the study participants. [file 1472-6947-14-30-S3.pdf]

ANNEX III - Additional quotations from the study participants

A Qualitative Evaluation of General Practitioners' views on Protocol-Driven eReferral in Scotland

Matt-Mouley Bouamrane & Frances S. Mair

1 SCI Gateway: System & Information Quality

1.1. SCI Gateway System Usage Patterns:

The following interview excerpt illustrates ad-hoc 'work-around' for eReferral management which has been implemented in one specific practice. GPs in the practice dictate clinical letters in a digital recorder which uploads the file to the practice admistrative staff, who then type these documents and fill in the SCI Gateway form. GPs then review the referral document before sending on the electronic referral via the SCI Gateway system:

- **GP25**: *"there's a paper-based system for tracing referrals within the practice with a... a printed list where you write the name of the patient and their local identifier, the sort of EMIS number* (the practice electronic medical record system) *and the date you've made the referral and then it goes... that goes with the tape that you've dictated through to administration and they fill the date that they've typed it up and then it will go for checking and because we're all part-time, referrals are not checked by the GP that created them"*

Interviewer: "O.K. So they're checked by another GP or...?"
GP25: *"...They're checked by another GP because otherwise we cannot have a 4 or 5 day delay in the referral being sent... So this piece of paper that went with the tape record: when it was checked, who checked it and what the date was and that completed sheet would then go back to the original GP who created it so that they can be certain that their referral has*

been finished off and sent... because we had a few instances where it fell through the... the referral fell through the net... and didn't get sent and were... so we created a new in-house process to make sure that things got checked... it allows a degree of security in the... O.K. it's based on bits of papers but..."

1.2. SCI Gateway - support for automatic data entry:

- **GP2**: *"...I type in my clinical details and provision as I would normally and then I would control C to copy and I would paste that in, so it's really quick. It pulls in all the past history, drug history, blood... you know: all the important information."*
- **GP13**: *"it usually links up the patient's details quite well so... you know... you are able to get all the patient details from your GP record transferred on to SCI Gateway, umhhh... quite well"*
- **GP16**: *"...I dictate the clinical details and the computer system picks of a lot of the details through the referral and so by the time it comes back to me it's been done by my secretary so it's ready to go"*

1.3. SCI Gateway - Support for providing additional clinical information:

Interviewer: "...What sort of information do you include in the referral letter that you send through SCI?"

- **GP1**: *"...what I... It will say very briefly what the patient has complained of but will say very briefly what I think might be wrong. I will say what it is what I'm looking for. And I will try and fill in some sort of contextual details for my acute colleagues who will probably not know the patient. You know so, I mean if this is somebody who is hugely anxious about life and things, I will say: 'this is a hugely anxious patient'. You know, just so that they've got some sort of vague inkling of the person behind the symptom complex"*
- **GP7**: *"The kind of things that are auto...populated, so the demographic information, there's a pass med... some past medical history and drug history and then really the history of presenting complaints and the presenting*

complaint and I usually like to put what I think the preferred management would be assuming that I have any idea. and I also like to correlate that back to what actually happens... whether my question has in fact been answered."

1.4. Administratively Cumbersome:

(when asked about the SCI protocol-driven referrals):

- **GP15**: *"...it's only helpful in the sense there that, you know... often those kinds of referrals are done after the patient has left the room so that'll all be flagged up: 'have you done this and have you thought about that?' which are helpful yeah, but for the most part the patient's been in a couple of hours ago so, you know... that's not helpful then because if he's not there and you haven't done it: do you have to get the patient back in? or do you have to put some sort of comments? so... pros and cons [...] It wouldn't be feasible for GPs to do that all the time because it requires some knowledge of IT and typing skills which you just don't have the time during the consultation to do or after I would think"*
- **GP25**: *"...the biggest challenge we have in Lothian is knowing which hospital provides which service... there's not a concept of the way of making a referral is: 'I make the decision that I need to refer somebody to um... gynaecology or you know some throat' so... but the paging sequence for SCI Gateway is to chose the hospital before you chose the specialty... so you've got to then go and look-up quickly which hospital offers ENT so, then pick that hospital, then pick ENT. From my point-of-view, the logical thing would be to pick the specialty first and then be offered a list of possible locations the patient might want to go to..."*
"... a lot of departments just use the basic SIGN referral structure referral and they're very quick and you don't need to do that while the patient is there whereas others you... you have a series of specific questions but they will answers to... sometimes you don't remember to ask them when the patient's there and sometimes you haven't so the easiest solution to make sure you don't miss anything is to start the referral with the patient's there, so to speak... that just take a bit longer than the 10 minutes you necessarily got to actually park it... My own personal approach has been to try to start the referral with the patient there so by the end of the surgery I can then go into SCI Gateway and have a look and see... you know, that's my

method of making a log of who needed referring"

1.5. Issues of System Performance:

- **GP1**: *"...apart from the time taken to open up SCI Gateway, and then to log into the referral screen and then each referral takes 8 mouse clicks to actually send. Now that... whoever dreamed that up was not a busy GP."*
- **GP6**: *"...And it takes... it's quite... slow once it's sort of try and start and submitting it, you know, it's not very quick. I can touch type so it would be just as quick for me to just type them as I am going along but I think that the internet is too slow and... I think it's probably the internet rather then SCI Gateway itself... it's just too slow and it's a waste of my time, I'm quicker to dictate"*
- **GP7**: *"Not easy. And again really too time consuming I would say. We use our receptionist... our typist use the SCI Gateway template. I've... couldn't spare the time to do them myself. [...] the templates aren't terrible easy, but I mean the speed of the website makes it really very difficult, takes about two or three times the length of time it would to dictate it."*

1.6. Information Presentation & System Status:

- **GP4**: *"...one of the secretaries [...] does keep an eye on referrals so, because some doctors... what you can do is that you can complete it, half of it and not send it. Now, I never do that 'cause I'm only in practice just once a week and I always do the whole thing and send it off... It's a bit frustrating because you have to send it and then you have to go back into the journal part of the note and click another button to proceed it, which again I think is unnecessary but, umh... what I was talking about being a bit, you know clunky with all the different things you've got to click... some GPs will leave it half-finished and come back to it and the secretary keeps an eye on whether some of them are sitting on the system that haven't gone so she nags at people to tell them to get them sent..."*

2 SCI Gateway: Information Usage & User satisfaction

2.1. Immediate Transfer of the Referral Request:

- **GP3**: *"...Och yeah, I mean, I think they go much quicker. I mean, I think you can get them away much quicker and it's you know... As soon as you press the button they're with the hospitals, so you know, you don't have the delay of the letter... the post..."*
- **GP19**: *"Well you get the same but everything's happening much quicker. I mean sometimes the letters may be lying a day or two before they were actually put onto the system and sent. So it feels you know... the perception is that you're doing it right away"*
- **GP25**: *"... The other big advantage is the speed with which referrals are moved because obviously you're not relying on bits of papers flying around in the internal system"*

2.2. Perceived Dis-Benefits:

- **GP1**: *"...I mean, each of these clicks must be important for some reason in the process. Nobody's kind of thought through the fact that each of these clicks then adds to each of the other clicks and creates a big... [...] So I mean even, you know, even shaving off a couple of minutes here and there would be wonderful."*
- **GP4**: *"...I find it frustrating, a little bit frustrating sometimes, there's certain boxes you've got to tick before you can send it off, so there's certain things like you've got to click on 'YES' or 'NO' for transport whereas it would be easier if just 'NO' was the default and if you wanted transport you would go and change it to 'YES' you know... [...] I find it that it takes longer than needs be. You feel you have to click, click, click, click through a few things each time you're making a referral which can be a bit..."*
- **GP24**: *"...generally I'll have dictated the letter and then when I come to check it... check it over, I have to fill in the protocol, which just really duplicates what's in the letter. So you have to re-enter individual fields [...] and each protocol is different so I can't really remember which specific*

question each particular specialty asks for [...] I mean, if I was doing the referral myself I would probably just do the protocol without writing very much in the letter but... uhhh, when I am pushed... when I'm struck for time, I do a letter and have the protocol but usually just answer 'as over' or 'see letter' or something..."

3 SCI Gateway: Individual & Organisational Impact

3.1. Improved Use of standard protocols and guidelines:

- **GP5**: *"Some of the referrals almost have in-built guidelines in order to actually do them, so they will say, they will request for example that certain bloods are being done or that the referral meets certain criteria so in some ways that is a modified guideline"*
- **GP6**: *"[...] I think the ones just now are good... And they can act as prompts so like for example when you're referring to obstetrics and gene(cology), there's one that asks you when their last meals was and I think sometimes you forget to give that information, and it's fair enough that they ask for that"*
- **GP15**: *"I think it gets probably, if you've done it once or twice and it keeps coming up with the same thing you're likely to remember the 3rd or 4th time when you're in consultation to do those things, yeah"*

3.2. Improved Organisational Work Processes and Performance:

- **GP15**: *"Most of the times, I dictate them and then the secretary do them up and then they come back to us to look at the screen and send on... some of us, I... I do occasionally... I'll do it for radiology ones and I'll do it for things that are urgent... I can type... I'll type it up myself and do it myself"*
- **GP22**: *"I mean, I do say to patients if they haven't heard with regards to an appointment within whatever amount of time I think is appropriate, it might be a month... if you haven't heard anything then get back to us*

and we'll chase up exactly were you are in the system... And we are doing quite a lot of that..."

Interviewer: "Are you? Yes? You do a lot of chasing up of... referrals?"

"Yeah... I mean, I must say it's much better now since, since we're using the SCI Gateway and there's a much easier a... you know, trail to follow. I mean going back 5 years before we were using SCI, patients were waiting for an appointment coming in and saying 'we haven't heard anything, we've phoned up to find out, or we've never received a letter' and then you're faxing the letter through again but obviously all these problems have been more or less... resolved completely since... the SCI Gateway, yes"

- **GP25**: *"...you also have the advantage that you can track the referral, you know that it's gone and that it's been received whereas with a bit of paper, once you've put in the envelope and it leaves the practice, there's no tracking on it... So those are useful features in terms of at least knowing that it safely got to the other end. I think that, as yet, there isn't the possibility to know how far through it is in terms of hospital triage and all that sort of system but..."*

- **3.3. Improved Information sharing / Systems Integration accross the Health Services**:

- **GP2**: *" Yeah, I mean, SCI Gateway is great... you make your referral you see that it's submitted, you can actually tell when someone's been appointed... if you look on the screen... so if they've come back in and they've not heard anything, you can have a look into that and see whether, you know, kind of what... whether it's been viewed, whether it's been appointed. It saves a lot of time, in the old days you'd have to phone up the secretaries, appointment people and try and find out what was happening, it was very difficult [...] You can see that if you go onto your SCI Gateway screen, you can tell what's happening [...] whether it's been viewed,* (i.e. the referral document) *...whether it's appointed. They don't give you an appointment date, but you can tell that... it says 'appointment', 'appointment' comes up so presumably that means they're in the system to get their appointment"*

- **3.4. Perceived Dis-Benefits**:

Electronic referral is more time-consuming or complex than previous practices: Although, several GPs did suggest that completing the electronic referrals could be more complex or time consuming than just dictating and signing referral letters, several also suggested that the benefits to the health system clearly outweighed the necessary overheads introduced by the system.

- **GP1**: *"...Well we.... I dictate the referral and my secretary types it onto the SCI referral form and then it's appears in the SCI Gateway for me to proofread and then go through the obligatory 8 clicks and about half a minute to send the thing. It is a rather tedious, slow process... it was much quicker to sign the letter."*
- **GP12**: *"I reckon..... I mean don't think we can re-wind the clock back but I reckon in some senses, you know, we used to dictate a letter, which we still do and used to get a draft form type stuff, which we often if it was correct, we'd just sort of print it and sign it type stuff it was quicker, the SCI is a little bit slower than that there."*
- **GP17**: *"I like it. It can sometimes take a little bit of time to complete the referral, but generally I think it's much better to refer to through SCI Gateway electronically."*

- **3.5. eReferral is designed to suit the information needs of the recipient of the referrals**:

- **GP12**: *"[...] From a primary care perspective, sometimes it seems a bit of a pain [...] to fill stuff in, so as they can decide what they want to do. And I suspect the stuff they ask you for is things that make a difference for the patients and helps the hospital. And in some sense, that's the perspective that we should have, you know, it's going to try and standardise the care that's offered to the patients and so as when you sort of press the send button, all that information goes to the hospital. And at the hospital they will respond to that [...]"*
- **GP23**: *" Well there's templates you use to fill in... every referral, there's a template. And the templates have been... they've been customised to the people that your sending them to, so they have decided what they want you to put in it."*

• **GP25**: *"...some of them* (i.e. referral protocols) *are very useful, some of them I feel get in the way, and you feel you're just duplicating information that you would have put in the medical history section in the sort of... your referral anyway [...] ...I can see that at the triage end, there is sometime benefit in prompting GP to – so that's from the hospital end – in prompting the GP to answer the GP to answer certain specific questions in part of the referral to make it easier to decide or... urgent or whatever it is to see that referral but sometimes, I do feel it just gets in the way and often it's getting in the way of the consultation because I am having to start the referral during the consultation which just takes that few seconds longer um... especially if I know, if I can remember that there's a significant protocol on it."*

- **3.6. Lack of Feed-back on referrals**:

• **GP3**: *"...I am coming around to thinking it would be quite useful for us to be notified as well. [...] because it seems to be one of the major problems we seem to have is in the area, is the problems with the postal service not delivering letters. [...] So we're then having to send another letter so that's another appointment wasted. And I think as an electronic system, whereby when the patient was appointed, we automatically got notification, I think that could help."*
"...you have to just phone up the secretary" (at the hospital)
• **GP6**: *"Um... Honestly, I actually don't know I think it seems to be sometimes the patients get a letter saying that they are... you know the hospital's received a referral and he will be contacted in due course for an appointment and then you know, it all sort of goes from: sometimes the patients are just given an appointment for tests for investigations without even being seen at the hospital clinic, it just depends on the clinical situation"*
Interviewer: how are you kept informed of how the referral is progressing?
"We're not kept informed as far as I can see. I think you could go on to SCI Gateway and you could see that it has been looked at, but I don't know that... beyond that, this been allocated and quite often you don't know that a patient's been to hospital until the patient comes back or the letter arrives"

• **GP7**: *"[...] if somebody comes in 12 weeks after you've referred them and said that they haven't heard anything... the only way of confirming that they haven't just lost the referral is to get somebody to phone up and check that they've received it. And you know, if I knew that there was no question of a referral being missed – which you think given that it's an electronic system wouldn't be too difficult to do – I wouldn't have any great problem about not having a confirmation of receipt of the... of the referral. We do get read receipts of the... when we send this guy for referral so we can see that it's being read. Problem is that may then have been lost, so that being read isn't any proof that they've actually going to action it."*

• **3.7. Lack of Coordination across the Health Services & Lack of work practice Coherence**:

Interviewer: "Do you use any pathways, referral pathways, guidelines?"
• **GP18**: *"Yes, we do, we have those really for cancer care pathway, for urgent referral pathways for certain specialities in cancers. That's quite commonplace. And recently there have been some pathways set up for orthopaedic, for spinal problems and knee problems."*
Interviewer: "So do you find that that there are sufficient guidance on referrals or do you think there are areas which aren't so clear?"
"Yes they are cumbersome... I think they have to be refined and they actually build in some extra steps which I think are unhelpful. [...] we now refer to a physiotherapy hub, so called, and they make a decision about referring on directly to orthopaedics or arranging scans or investigating prior to the orthopaedic appointment. And I think GPs could actually do that ourselves, if we had direct access to the investigative procedures [...] if we had direct access to the imaging process that would actually enhance the process even more so [...]"
